# Temperature of the Kenhawa Salt Wells

**Published:** 1847-10

**Authors:** 


*Temperature of the Kenhawa Salt Wells.*—It has been frequently stated, that the salt wells of Kenhawa do not conform to the law of increasing temperature with descent towards the interior of the earth. A correspondent made a series of observations on this point a few months since, and the result is that the water of those wells is several degrees warmer than that of the springs in the neighborhood. For example, he found the temperature of some of the deepest wells 63 degrees, while the mean temperature of several springs around was 48. The depth of the deepest well was 900 feet.—*Ibid.*

